# Nasal saline irrigation in preschool children: a survey of attitudes and prescribing habits of primary care pediatricians working in northern Italy

**DOI:** 10.1186/1824-7288-40-47

**Published:** 2014-05-15

**Authors:** Paola Marchisio, Marina Picca, Sara Torretta, Elena Baggi, Angela Pasinato, Sonia Bianchini, Erica Nazzari, Susanna Esposito, Nicola Principi

**Affiliations:** 1 Pediatric Highly Intensive Care Unit, Department of Pathophysiology and Transplantation, University of Milan, Fondazione IRCCS Ca’ Granda Ospedale Maggiore Policlinico, Via Commenda 9, 20122 Milano, Italy; 2 Primary care pediatrician, Milan, Italy; 3 Otolaryngology Unit, Department of Clinical Sciences and Community Health, University of Milan, Fondazione IRCCS Ca’ Granda Ospedale Maggiore Policlinico, Milan, Italy; 4 Primary care pediatrician, Torri di Quartesolo, Vicenza, Italy

**Keywords:** Isotonic saline solution, Hypertonic saline solution, Nasal saline irrigation, Nasal spray, Respiratory tract infection

## Abstract

**Background:**

It has been shown that nasal saline irrigation (NSI) alone can be effective in children with infectious and/or allergic respiratory problems, but no study has assessed the awareness or clinical use of NSI among practising pediatricians. The main aim of this study was to evaluate the use of NSI in pre-school children by primary care pediatricians working in northern Italy.

**Methods:**

Nine hundred randomly selected National Health Service primary care pediatricians with an e-mail address were sent an e-mail asking whether they were willing to respond to a questionnaire regarding the use of NSI. The 870 who answered positively were sent an anonymous questionnaire by post and e-mail that had 17 multiple-choice items.

**Results:**

Completed questionnaires were received from 860 of the 870 primary care pediatricians (98.8%). NSI was used by almost all the respondents (99.3%), although with significant differences in frequency. It was considered both a prophylactic and a therapeutic measure by most of the respondents (60.3%), who prescribed it every day for healthy children and more frequently when they were ill. Most of the primary care pediatricians (87%) indicated an isotonic solution as the preferred solution, and the most frequently recommended administration devices were a nasal spray (67.7%) and bulb syringe (20.6%). Most of the pediatricians (75.6%) convinced parents to use NSI by explaining it could have various beneficial effects, and two-thirds (527/854; 61.7%) thought that most of the parents agreed about the importance of NSI. Analysis of possible associations between NSI prescribing behaviour and the demographic data revealed an associations with age and gender, with pediatricians aged <50 years prescribing NSI more frequently than their older counterparts (p < 0.01), and females prescribing NSI more frequently than males (p < 0.01).

**Conclusions:**

In Northern Italy, most primary care pediatricians prescribe NSI for both the prophylaxis and therapy of upper respiratory tract problems in pre-school children. However, many aspects of the procedure are not clarified, and this reduces parental compliance. Given the medical and economic advantages of NSI, this situation should be changed as soon as possible.

## Background

A number of recent studies have shown that nasal saline irrigation (NSI), a practice widely used to treat upper respiratory tract diseases in adults [1,2], can also be effective in children with infectious and/or allergic respiratory problems not only as adjunctive treatment, but also alone [3-8]. NSI significantly reduces nasal secretions/post-nasal drip in children with chronic rhinosinusitis, and considerably improves radiographic signs of disease [3]; it also reduces the need for surgery even in patients resistant to medical treatment with antibiotics and nasal corticosteroids [4,5]. Although it is not effective in reducing inflammation in nasal smears or modifying post-treatment radiography when prescribed to children with acute rhinosinusitis, it does improve mean quality life scores by reducing nasal symptoms and significantly increasing peak nasal expiratory flow [6]. Finally, Garavello *et al*. [7] and Marchisio *et al*. [8] have found that it reduces signs and symptoms of seasonal allergic rhinitis and limits the need for antihistamines. All of these studies showed that NSI is safe and well tolerated because there were no severe adverse events and only a minority of children had to discontinue the treatment because of poor tolerance [3-8]. It has also been shown that the procedure is quite inexpensive and reduces the use of prescription and over-the-counter medications, and therefore have a substantial impact not only on medical costs, but also on antibiotic pressure and the associated antibiotic resistance [9].

On the basis of these findings, a number of experts have identified NSI as an appropriate adjunctive treatment for many pediatric upper respiratory tract diseases, and some scientific societies have included it in their treatment guidelines for selected respiratory diseases [10,11]. However, it is not clear how or how extensively NSI is used in everyday practice, particularly in the community and in younger children. The only available data, which were collected some years ago from family physicians in Wisconsin [12], indicate that NSI is frequently prescribed for a variety of upper respiratory conditions, but administered using various dosing schedules and types of solution, some of which are different from those suggested in the studies that have found the practice effective, safe and well tolerated. No study has assessed the awareness or clinical use of NSI among practising pediatricians, and so nothing is known about the extent of their awareness of the procedure, how they use it and for what conditions, its clinical successfulness or otherwise, or the physician characteristics that might influence their NSI-related practice patterns.

Main aim of this study was to evaluate the use of NSI in pre-school children by primary care pediatricians working in northern Italy.

## Methods

### Study design

This cross-sectional survey of the use of NSI by a representative sample of primary care pediatricians working in Northern Italy was carried out between 10 January 2012 and 31 March 2013. The study was approved by the Ethics Committee of the University of Milan, Italy, and informed consent was obtained from all of the participants before study entry.

### Study population

A group of 900 National Health Service primary care pediatricians with an e-mail address were randomly selected by means of a computer-based randomisation list from among those working for the in the northern regions of Italy (Piedmont, Liguria, Lombardy, Veneto, Friuli-Venezia Giulia and Emilia-Romagna). In order to assure a fully representative sample, the same percentage of pediatricians was selected in each region. The physicians were sent an e-mail before the beginning of the survey in which they were asked whether they were willing to respond to a questionnaire regarding their use of NSI. The 870 pediatricians who answered positively were e-mailed an anonymous questionnaire that was also sent by post together with a stamped envelope addressed to the trained study researchers (PM, ST, EB and SB). Fifteen days later, any pediatrician who had not returned the completed questionnaire was telephoned and urged to do so.

### Questionnaire design and administration

The questionnaire, which was anonymous but coded in order to be able to identify non-responders and ensure the elimination of multiple responses, was conceived by the first author (PM) in collaboration with the co-authors (MP,AP) and pilot tested on a sample of 20 pediatricians in Milan, Italy. It required about 10 minutes to complete and guided respondents through 17 multiple-choice items divided into two main sections: one for personal and demographic data, including gender, and the years of birth, graduation and specialisation; the other concerning attitudes towards the use of NSI in pre-school children (i.e. personal opinions about the efficacy and usefulness of NSI) and the prescribing behaviour adopted in routine clinical practice (i.e. indication, frequency of administration, the composition of the solutions, administration method).

### Statistical analysis

The data were descriptively analysed to assess the prevalence and distribution of all the variables. The continuous variables were expressed as mean values and standard deviation (SD), and the categorical variables were expressed as absolute numbers and percentages. The categorical variables were analysed dichotomously and at multiple levels. The Kruskal-Wallis equality-of-populations rank test and Fisher’s exact test were used to determine whether the attitudes toward NSI and prescribing behaviours were related to the demographic data. Simple and multiple logistic regression models were used after adjusting for the main confounders; and odds ratios (ORs) and their standard error (SE) and 95% confidence intervals (95% CI) were computed to measure the strength of the associations. Statistical significance was set at p = 0.05. The data were analysed using STATA 10.0 software (StataCorp, College Station, TX).

## Results

Questionnaires were completed by 860 (98.8%) of the 870 primary care pediatricians, most of whom were female (635; 73.8%), aged >50 years (557; 64.8%), and had been practising as primary care pediatricians for more than 20 years (509; 59.1%) (Table 1).

**Table 1:** Demographic characteristics of the primary care pediatricians who returned completed questionnaires concerning their use of nasal saline irrigation (NSI)

**Demographic characteristics**	**No. of primary care pediatricians**	**Percentage**
Total number	860	
Females	635	73.8
Age, years		
>50	557	64.8
35-50	273	31.7
<35	13	1.5
Non-responders	17	2.0
No. years since graduation		
>30	178	20.7
20-30	495	57.5
10-19	132	15.3
<10	30	3.5
Non-responders	25	2.9
No. of years since specialising in pediatrics		
>30	77	8.9
20-30	432	50.2
10-20	243	28.2
<10	83	9.6
Non-responders	25	2.9

Table 2 shows that almost all the pediatricians prescribed NSI for pre-school children (854/860; 99.3%), although with significant differences in frequency: it was prescribed at least once to >75% of their younger patients by 358 (41.9%), to 25-75% by 452 (52.9%), and to <25% by 44 (5.2%). About 45% of the respondents considered NSI important for patients of all pre-school ages, whereas 14.2% thought that it was more important for children aged 2–3 years, and 37.8% that it was more important for those aged <1 year. Most of the respondents (60.3%) considered NSI both prophylactic and therapeutic. Of the 515 pediatricians who also prescribed it for prophylaxis, 84.5% recommended its administration 3–4 times a week whereas, in the case of ill children, the frequency of administration was once daily (37.1%), 2–3 times a day (48.2%), or even more frequently (14.7%). Most of the primary care pediatricians (87%) indicated isotonic solution as the preferred solution, and only 7.8% recommended hypertonic solutions. The most frequently recommended methods of administration were nasal sprays (67.7%) followed by the use of a bulb syringe (20.6%). As regards the volume of solution, 28.2% suggested 5–20 mL per nostril regardless of age, 23.0% adjusted the volume on the basis of age, and 20.7% did not prescribe more than 2.5 mL per nostril.

**Table 2:** Primary care pediatricians’ use of nasal saline irrigation in pre-school children

**Parameter**	**Possible answers**	**No. of primary care pediatricians**	**Percentage**
Percentage of pre-school children for whom NSI is recommended			
	None	6/860	0.7
	<25%	44/860	5.1
	25-75%	452/860	52.5
	>75%	358/860	41.6
Age of patients for whom NSI is considered important			
	All pre-school years	473/854	43.7
	<1 year	323/854	37.8
	1-2 year	37/854	4.3
	2-3 years	121/854	14.2
Use of NSI for upper respiratory tract diseases			
	Treatment of acute phase	339/854	39.7
	Prophylaxis in healthy children	515/854	60.3
Frequency of therapeutic administration of NSI			
	Once per day	317/854	37.1
	2-3 times per day	412/854	48.2
	>3 times per day	125/854	14.7
Frequency of prophylactic administration of NSI			
	Never	339/854	39.7
	Daily	80/515	15.5
	3-4 times per week	435/515	84.5
Type of solution			
	Isotonic saline solution	743/854	87.0
	Hypertonic saline solution	67/854	7.8
	Hypotonic saline solution	44/854	5.2
Method of administration			
	Spray	578/854	67.7
	Bulb syringe	176/854	20.6
	Gravity	100/854	11.7
Volume of solution recommended for NSI			
	Depending on patient age	196/854	23.0
	5-20 mL per nostril	241/854	28.2
	5 mL per nostril	157/854	18.4
	2.5 mL per nostril	177/854	20.7
	Other	83/854	9.7
Final evaluation of efficacy and safety of NSI			
	Effective and safe	845/860	98.3
	Effective but poorly tolerated	9/860	1.0
	Ineffective	2/860	0.2
	No opinion	4/860	0.5

Table 3 shows how the primary care pediatricians instructed parents to administer NSI and the reasons for parental refusal to use it. Parental education was most frequently only verbal (54.5%), whereas 33.2% of the pediatricians gave a practical demonstration, and 10.4% written instructions; only 1.9% gave no instructions at all. Most of the pediatricians (75.6%) convinced parents to use NSI by explaining it that it had a number of beneficial effects, including improved nasal respiration, a reduction in the bacterial complications of viral respiratory infection, and a reduction in the duration of viral illnesses; a few pediatricians cited only one of these advantages, and three (0.3%) did not give any explanation. Concerning the direction in which to move the syringe or nozzle of the device used to administer NSI, 33.0% declared that they did not suggest any direction, whereas 29.4% and 23.6% respectively recommended “toward the ipsilateral ear” and “toward the contralateral ear”. Two-thirds of the prescribing pediatricians (527/854; 61.7%) thought that most of the parents of their patients agreed that NSI was important, but the main perceived reasons for parental refusal were the difficulty of administration (471; 55,1%) or the supposed invasiveness of the procedure (279; 32.7%).

**Table 3:** Parents’ NSI education by primary care pediatricians, and judgement of parents’ compliance

**Parameter**	**Possible answer**	**No. of primary care pediatricians**	**Percentage**
Method used to educate parents			
	Verbal instructions	465/854	54.5
	Written instructions	89/854	10.4
	Practical demonstration	284/854	33.2
	No instructions	16/854	1.9
Reasons given to convince parents to use NSI			
	Improved nasal respiration	70/854	8.2
	Reduced bacterial super-infection	90/854	10.5
	Improved treatment of respiratory infection	29/854	3.4
	All of these reasons	645/854	75.6
	Other	17/854	2.0
	No reason	3/854	0.3
Direction in which to move the administration device			
	Toward the ipsilateral ear	250/854	29.4
	Toward the contralateral ear	202/854	23.6
	Other	120/854	14.0
	No suggestion	282/854	33.0
Recommended position for NSI			
	Infants: lying on one side; Older children: bending forward over a sink with the head tilted down and a little to one side	639/854	74.8
	On the side at any age	146/854	17.1
	Other	35/854	4.1
	No suggestion	34/854	4.0
Reasons parents are not compliant with recommendation to use NSI			
	Not useful	194/854	12.2
	Dangerous	279/854	32.7
	Difficult to administer	471/854	55.1

**Figure 1:**
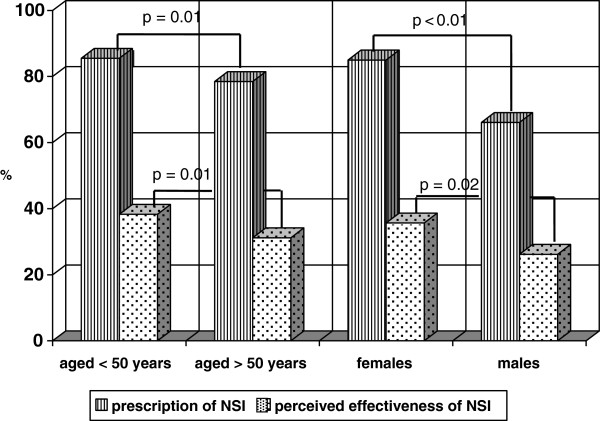
Proportions of pediatricians prescribing NSI and considering NSI effective by age and gender.

Analysis of the possible associations between NSI prescribing behaviour and the demographic data revealed that the number of patients for which NSI was prescribed and judgements of its efficacy were apparently influenced by age and gender. Pediatricians aged <50 years prescribed NSI more frequently than their older counterparts (85.5% *vs* 78.5%; p = 0.01), and females prescribed NSI more frequently than males (85.0% *vs* 66.1%; p < 0.01) (Figure 1). Moreover, younger pediatricians considered NSI effective more frequently than the older pediatricians (38.3% *vs* 31.2%; p = 0.01), and females more frequently than males (35.7% vs. 26.2%; p = 0.02). However, multiple logistic regression analysis showed that only gender adjusted for age remained significantly associated with the prescription of NSI (OR = 2.76, SE = 0.53, 95% CI 1.89-4.04, p < 0.01) and a positive opinion concerning its usefulness (OR = 1.52, SE = 0.28, p = 0.03). Moreover, parents understood the importance of NSI significantly more frequently when their pediatricians considered NSI effective (63.0% *vs* 49.0%; p < 0.01). None of the other demographic variables was statistically associated with attitudes toward NSI or prescribing behaviour.

## Discussion

This is the first study designed to evaluate primary care pediatricians’ knowledge and prescription of NSI in pre-school children. The randomised selection of potential participants and the fact that the number of those who refused to take part in the survey was very small makes it unlikely that the responders were only those aware of NSI. Consequently, it is reasonable to conceive that the study population was truly representative of the primary care pediatricians living in Northern Italy and working for the National Health Service.

The data indicate that, despite some age- and gender-related differences, the majority of the respondents knew NSI, prescribed it for their pre-school patients, and considered it effective and well tolerated. As NSI is a relatively new means of treating upper respiratory problems in younger children, it is not surprising that younger pediatricians were more likely to use it and consider it effective than those aged >50 years. The use of NSI in younger patients is not extensively examined in the scientific literature because most pediatric studies of NSI have mainly involved school-age children . However, Rabago *et al*. [12] found that family physicians in Wisconsin included children aged <7 years among the subjects eligible for NSI. The opinion of these physicians concerning the tolerability of NSI is similar to that of the primary care pediatricians enrolled in this study, and is supported by the data collected in other studies evaluating the effect of NSI on children [3-8]. The incidence of adverse events following NSI was usually very low, and most of the studies highlighted the fact that they were not severe enough to preclude continuing treatment.

A substantial proportion of the respondents prescribed NSI not only for the treatment of rhinitis and upper respiratory tract infections, but also for prophylactic purposes. The widespread therapeutic use of NSI was not surprising given the frequency of these diseases in younger children, their very high tendency to recur, the positive opinion of the pediatricians concerning the effectiveness of NSI, and the results of the published pediatric studies. On the contrary, its prophylactic use was quite unexpected because NSI has never been evaluated in randomised, double-blind and placebo-controlled studies.

The only published studies are two open studies. In the first, children aged 6–10 years with uncomplicated cold or influenza were treated with NSI and standard therapy or NSI alone for three months, and the cure of the first episode and any subsequent recurrences were recorded [13]. Nasal symptoms during acute illness resolved more rapidly in the children treated with NSI alone, who also experienced less frequent recurrences of rhinitis. The second was a Russian multi-centre, open-label, randomised study, which found that NSI reduced the morbidity due to acute respiratory infections in children attending secondary schools and day-care centres by 2.4-3.2 times throughout the epidemiological period, and simultaneously improved the clinical course of upper respiratory tract diseases and bronchial asthma [14].

However, some of the presumed mechanisms of action of NSI may explain why our primary care pediatricians think it effective as preventive measure. In addition to cleaning the nasal cavities and removing antigens and local inflammatory mediators such as histamine and prostaglandins, it is thought that NSI may improve mucus clearance by enhancing ciliary beat frequency, thus reducing the risk of bacterial super-infections and enhancing mucosal healing [15]. This may be more beneficial during the winter (when respiratory infections are more frequent) because of the co-existence of conditions related to impaired respiratory epithelial ciliary activity, such as low temperatures, air pollution, inspired air humidity and dehydration [16].

The most favoured way of administering NSI was by means of a nasal spray, whereas only about 20% of the respondents recommended a bulb syringe. The most appropriate method of administration is still subject to debate. A review published in 2010 found that high-volume, positive-pressure devices led to better fluid distribution throughout the sinuses than low-volume applications such as nebulisers or sprays, or low-pressure devices such as the Neti pot [17]. However, it only considered adult studies, and there are no published data comparing bulb syringes and sprays in children, particularly very young subjects. There is therefore a need for pediatric studies but, in meantime, it can be suggested that NSI should be started using a bulb syringe because of the larger amount of solution it delivers, and that its use may be continued if the child tolerates it without any problem.

Most of the respondents use isotonic saline for a NSI, and only about 8% use hypertonic saline. This does not seem to be in line with the literature because a number of *in vitro*[18] and clinical studies [1,3,8], including pediatric studies [3,8], have found that hypertonic saline is more effective than isotonic in reducing the signs and symptoms of upper respiratory diseases. However, the effect of hypertonic saline has only been tested in ill patients, and the better results may be explained by its greater activity in improving mucociliary clearance [18]. Furthermore, it has not been demonstrated that hypertonic saline is better in the case of prophylaxis, and it is worth remembering that it may be a little less tolerated because it can cause uncomfortable burning or stinging sensations, even if rarely [1]. Both solutions are able to clear germs, allergens and other pollutants from the nasopharynx and can protect children against respiratory diseases. Once again, further studies are needed, but it can be suggested that normal saline should be used for prophylaxis and hypertonic saline for therapy.

About 75% of the respondents chose the correct position for NSI: i.e. infants should be lying down on their side, whereas older children bend forward over a sink with their head tilted down and a little to a side in the older ones. This is not surprising because the correct position has been described on websites [19-22] and in newpapers and magazines [23]. This is also true for Italy, where the most important newspaper has repeatedly published the best way to use administer NSI to children in its health section [24].

On the contrary, the answers to the questions regarding the volume of liquid to use were disappointing because only about 25% of the pediatricians declared that it depended on the child’s age, and about 20% prescribed only 2.5 mL per nostril for all children. This highlights the poor knowledge of Italian primary care pediatricians and is probably due to the lack of precise data in information sources [19-24]. The same can be said about the responses concerning the direction of administration because only about 30% of the responders told parents to move the syringe or nozzle toward the ipsilateral ear; once again, there is a lack of adequate published information.

Most of the respondents declared that parents have generally understood the importance of NSI, and that was particularly evident when the pediatricians themselves were convinced of its efficacy. However, about 40% expressed doubts about parental compliance mainly because of a certain difficulty in administration or the supposed invasiveness of the procedure. These doubts seem to due to the lack of adequate information concerning the correct amount of solution and the best way of administering. The findings of this study seem to indicate, that if NSI is to be completely accepted by parents, it is essential that pediatricians clarify these points and communicate their conclusions to parents. Studies involving younger children and health authority educational programmes are urgently needed.

## Conclusions

Primary care pediatricians in northern Italy largely use NSI for prophylaxis and to treat upper respiratory tract problems in pre-school children. However, many aspects of the procedure have not been clarified and this reduces parental compliance. Given the medical and economic advantages of NSI, it is essential to change this situation as soon as possible.

## Abbreviations

95% CI: 95% confidence intervals; NSI: Nasal saline irrigation; ORs: Odds ratios; SD: Standard deviation; SE: Standard error.

## Competing interests

The authors declare that they have no competing interests.

## Authors’ contributions

PM designed the study and co-drafted the manuscript; MP and AP co-designed the study, enrolled the respondents; ST co-drafted the manuscript and made the statistical analysis; EB, SB and EN were responsible for data management; SE and NP critically reviewed the intellectual content of the manuscript, re-wrote part of the text, and approved the version to be published. All of the authors read and approved the final manuscript.
